# 2530. Pharmacokinetic-Pharmacodynamic (PK-PD) Target Attainment Analyses to Support Ceftobiprole Dose Selection for the Treatment of Patients with Acute Bacterial Skin and Skin Structure Infections (ABSSSI) and Community-Acquired Bacterial Pneumonia (CABP)

**DOI:** 10.1093/ofid/ofad500.2148

**Published:** 2023-11-27

**Authors:** Sujata M Bhavnani, Jeffrey P Hammel, Anthony J Rinaldo, Jennifer Smart, Karine Litherland, Leonard R Duncan, Mark E Jones, Marc Engelhardt, Paul G Ambrose, Christopher M Rubino

**Affiliations:** Institute for Clinical Pharmacodynamics, Schenectady, NY; Institute for Clinical Pharmacodynamics, Schenectady, NY; Institute for Clinical Pharmacodynamics, Schenectady, NY; Basilea Pharmaceutica International Ltd, Allschwil, Basel-Landschaft, Switzerland; Basilea Pharmaceutica International Ltd, Allschwil, Basel-Landschaft, Switzerland; JMI Laboratories, North Liberty, Iowa; Basilea Pharmaceutica International Ltd., Allschwil, Switzerland, Allschwil, Basel-Landschaft, Switzerland; Basilea Pharmaceutica International Ltd., Allschwil, Switzerland, Allschwil, Basel-Landschaft, Switzerland; Institute for Clinical Pharmacodynamics, Schenectady, NY; Institute for Clinical Pharmacodynamics, Schenectady, NY

## Abstract

**Background:**

Ceftobiprole medocaril is an intravenously (IV) administered cephalosporin that is rapidly converted *in vivo* to the active moiety ceftobiprole. Ceftobiprole medocaril is being developed for the treatment of patients with ABSSSI and CABP. PK-PD target attainment analyses were performed to support the dosing regimens selected for these indications.

**Methods:**

Using a population PK model developed for ceftobiprole, PK-PD targets for efficacy, and *in vitro* surveillance data, percent probabilities of PK-PD target attainment were evaluated among simulated patients with ABSSSI and CABP resembling each clinical trial population. Dosing regimens were adjusted by CLcr group to match exposures among simulated patients with normal renal function administered ceftobiprole 500 mg IV every 8 hours. Randomly assigned ceftobiprole free-drug plasma %T > MIC (%*f*T >MIC) targets associated with net bacterial stasis and 1- and/or 2-log_10_ CFU reductions from baseline for *S. aureus* (SA), *Streptococcus* species, and Enterobacterales (ENT) based on data from a neutropenic murine-thigh infection model were assessed.

**Results:**

Selected ceftobiprole dosing regimens by CLcr group are shown in **Table 1**. For ABSSSI, the highest MIC values at which percent probabilities of PK-PD target attainment were ≥ 90% based on randomly assigned %*f*T > MIC targets associated with net bacterial stasis were ≥ 4 µg/mL for SA and *Streptococcus* species (inhibiting 100% of isolates) and ≥ 2 µg/mL for ENT (inhibiting 86.5% of isolates) (**Table 2**). For CABP, the highest MIC values at which percent probabilities of PK-PD target attainment were ≥ 90% based on %*f*T >MIC targets associated with a 1-log_10_ CFU reduction from baseline were ≥ 4 µg/mL for *S. pneumoniae* (inhibiting 100% of isolates) and SA and ≥ 2 µg/mL for ENT (**Table 3**). Using the same PK-PD target endpoints for each indication, overall percent probabilities of PK-PD target attainment (i.e., averaged over each MIC distribution) were > 99.9% for SA and *Streptococcus* species or *S. pneumoniae* and ≥ 98.7% for non-ESBL-producing ENT subsets.

**Figure:**
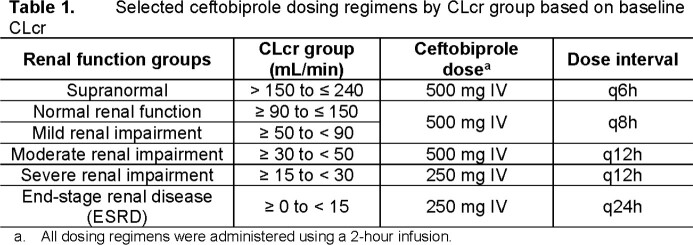

**Figure:**
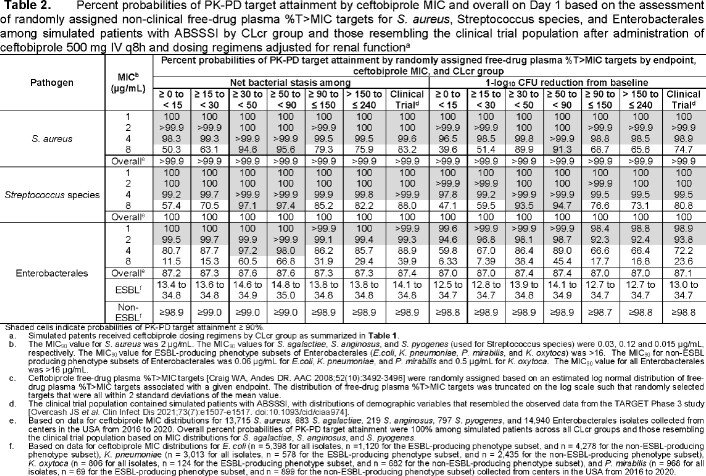

**Figure:**
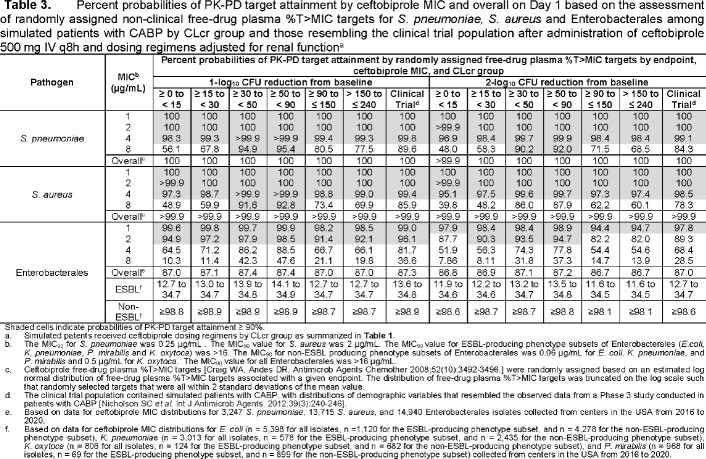

**Conclusion:**

These data provided support for a dosing regimen of ceftobiprole 500 mg IV every 8 hours for patients with ABSSSI or CABP. Dosing regimens should be adjusted for moderate and severe renal impairment including patients with ESRD.

**Disclosures:**

**Sujata M. Bhavnani, PharmD; MS; FIDSA**, Adagio Therapeutics, Inc.: Grant/Research Support|Albany Medical Center: Grant/Research Support|Amplyx Pharmaceuticals, Inc.: Grant/Research Support|AN2 Therapeutics: Grant/Research Support|Antabio SAS: Grant/Research Support|Arcutis Biotherapeutics, Inc.: Grant/Research Support|B. Braun Medical Inc.: Grant/Research Support|Basilea Pharmaceutica: Grant/Research Support|BioFire Diagnostics LLC: Grant/Research Support|Boston Pharmaceuticals: Grant/Research Support|Cidara Therapeutics Inc.: Grant/Research Support|Cipla USA: Grant/Research Support|Crestone Inc.: Grant/Research Support|CXC: Grant/Research Support|Debiopharm International SA: Grant/Research Support|Entasis Therapeutics: Grant/Research Support|Genentech: Grant/Research Support|GlaxoSmithKline: Grant/Research Support|Hoffmann-La Roche: Grant/Research Support|ICPD: Ownership Interest|Inotrem: Grant/Research Support|Insmed Inc.: Grant/Research Support|Iterum Therapeutics Limited: Grant/Research Support|Kaizen Bioscience, Co.: Grant/Research Support|KBP Biosciences USA: Grant/Research Support|Matinas Biopharma: Grant/Research Support|Meiji Seika Pharma Co., Ltd.: Grant/Research Support|Melinta Therapeutics: Grant/Research Support|Menarini Ricerche S.p.A.: Grant/Research Support|Mutabilis: Grant/Research Support|Nabriva Therapeutics AG: Grant/Research Support|Paratek Pharmaceuticals, Inc.: Grant/Research Support|Qpex Biopharma: Grant/Research Support|Sfunga Therapeutics: Grant/Research Support|Spero Therapeutics: Grant/Research Support|Suzhou Sinovent Pharmaceuticals Co.: Grant/Research Support|Theravance: Grant/Research Support|tranScrip Partners: Grant/Research Support|University of Wisconsin: Grant/Research Support|Utility Therapeutics: Grant/Research Support|ValanBio Therapeutics Inc.: Grant/Research Support|VenatoRx: Grant/Research Support **Jeffrey P. Hammel, MS**, Adagio Therapeutics, Inc.: Grant/Research Support|Albany Medical Center: Grant/Research Support|Amplyx Pharmaceuticals, Inc.: Grant/Research Support|AN2 Therapeutics: Grant/Research Support|Antabio SAS: Grant/Research Support|Arcutis Biotherapeutics, Inc.: Grant/Research Support|B. Braun Medical Inc.: Grant/Research Support|Basilea Pharmaceutica: Grant/Research Support|BioFire Diagnostics LLC: Grant/Research Support|Boston Pharmaceuticals: Grant/Research Support|Cidara Therapeutics Inc.: Grant/Research Support|Cipla USA: Grant/Research Support|Crestone Inc.: Grant/Research Support|CXC: Grant/Research Support|Debiopharm International SA: Grant/Research Support|Entasis Therapeutics: Grant/Research Support|Genentech: Grant/Research Support|GlaxoSmithKline: Grant/Research Support|Hoffmann-La Roche: Grant/Research Support|ICPD: Employee|Inotrem: Grant/Research Support|Insmed Inc.: Grant/Research Support|Iterum Therapeutics Limited: Grant/Research Support|Kaizen Bioscience, Co.: Grant/Research Support|KBP Biosciences USA: Grant/Research Support|Matinas Biopharma: Grant/Research Support|Meiji Seika Pharma Co., Ltd.: Grant/Research Support|Melinta Therapeutics: Grant/Research Support|Menarini Ricerche S.p.A.: Grant/Research Support|Mutabilis: Grant/Research Support|Nabriva Therapeutics AG: Grant/Research Support|Paratek Pharmaceuticals, Inc.: Grant/Research Support|Qpex Biopharma: Grant/Research Support|Sfunga Therapeutics: Grant/Research Support|Spero Therapeutics: Grant/Research Support|Suzhou Sinovent Pharmaceuticals Co.: Grant/Research Support|Theravance: Grant/Research Support|tranScrip Partners: Grant/Research Support|University of Wisconsin: Grant/Research Support|Utility Therapeutics: Grant/Research Support|ValanBio Therapeutics Inc.: Grant/Research Support|VenatoRx: Grant/Research Support **Anthony J. Rinaldo, B.S.**, Adagio Therapeutics, Inc.: Grant/Research Support|Albany Medical Center: Grant/Research Support|Amplyx Pharmaceuticals, Inc.: Grant/Research Support|AN2 Therapeutics: Grant/Research Support|Antabio SAS: Grant/Research Support|Arcutis Biotherapeutics, Inc.: Grant/Research Support|B. Braun Medical Inc.: Grant/Research Support|Basilea Pharmaceutica: Grant/Research Support|BioFire Diagnostics LLC: Grant/Research Support|Boston Pharmaceuticals: Grant/Research Support|Cidara Therapeutics Inc.: Grant/Research Support|Cipla USA: Grant/Research Support|Crestone Inc.: Grant/Research Support|CXC: Grant/Research Support|Debiopharm International SA: Grant/Research Support|Entasis Therapeutics: Grant/Research Support|Genentech: Grant/Research Support|GlaxoSmithKline: Grant/Research Support|Hoffmann-La Roche: Grant/Research Support|ICPD: Employee|Inotrem: Grant/Research Support|Insmed Inc.: Grant/Research Support|Iterum Therapeutics Limited: Grant/Research Support|Kaizen Bioscience, Co.: Grant/Research Support|KBP Biosciences USA: Grant/Research Support|Matinas Biopharma: Grant/Research Support|Meiji Seika Pharma Co., Ltd.: Grant/Research Support|Melinta Therapeutics: Grant/Research Support|Menarini Ricerche S.p.A.: Grant/Research Support|Mutabilis: Grant/Research Support|Nabriva Therapeutics AG: Grant/Research Support|Paratek Pharmaceuticals, Inc.: Grant/Research Support|Qpex Biopharma: Grant/Research Support|Sfunga Therapeutics: Grant/Research Support|Spero Therapeutics: Grant/Research Support|Suzhou Sinovent Pharmaceuticals Co.: Grant/Research Support|Theravance: Grant/Research Support|tranScrip Partners: Grant/Research Support|University of Wisconsin: Grant/Research Support|Utility Therapeutics: Grant/Research Support|ValanBio Therapeutics Inc.: Grant/Research Support|VenatoRx: Grant/Research Support **Jennifer Smart, PhD**, Basilea Pharmaceutica International Ltd, Allschwil, Switzerland: Stocks/Bonds **Karine Litherland, Ph.D.**, Basilea Pharmaceutica International Ltd, Allschwil, Switzerland: Full time employee|Basilea Pharmaceutica International Ltd, Allschwil, Switzerland: Stocks/Bonds **Leonard R. Duncan, PhD**, AbbVie: Grant/Research Support|Basilea: Grant/Research Support|CorMedix: Grant/Research Support|Melinta: Grant/Research Support|Pfizer: Grant/Research Support **Mark E. Jones, PhD**, Astellas Pharma Global Development, Inc: Support for the present publication|Basilea Pharmaceutica International Ltd: Employee of Basilea Pharmaceutica International Ltd|Basilea Pharmaceutica International Ltd: Stocks/Bonds **Marc Engelhardt, MD**, Astellas Pharma Global Development, Inc.: Support for the present publication|Basilea Pharmaceutica International Ltd: Employee of Basilea Pharmaceutica International Ltd|Basilea Pharmaceutica International Ltd: Stocks/Bonds **Paul G. Ambrose, PharmD; MS; FIDSA**, Adagio Therapeutics, Inc.: Grant/Research Support|Albany Medical Center: Grant/Research Support|Amplyx Pharmaceuticals, Inc.: Grant/Research Support|AN2 Therapeutics: Grant/Research Support|Antabio SAS: Grant/Research Support|Arcutis Biotherapeutics, Inc.: Grant/Research Support|B. Braun Medical Inc.: Grant/Research Support|Basilea Pharmaceutica: Grant/Research Support|BioFire Diagnostics LLC: Grant/Research Support|Boston Pharmaceuticals: Grant/Research Support|Cidara Therapeutics Inc.: Grant/Research Support|Cipla USA: Grant/Research Support|Crestone Inc.: Grant/Research Support|CXC: Grant/Research Support|Debiopharm International SA: Grant/Research Support|Entasis Therapeutics: Grant/Research Support|Genentech: Grant/Research Support|GlaxoSmithKline: Grant/Research Support|Hoffmann-La Roche: Grant/Research Support|ICPD: Ownership Interest|Inotrem: Grant/Research Support|Insmed Inc.: Grant/Research Support|Iterum Therapeutics Limited: Grant/Research Support|Kaizen Bioscience, Co.: Grant/Research Support|KBP Biosciences USA: Grant/Research Support|Matinas Biopharma: Grant/Research Support|Meiji Seika Pharma Co., Ltd.: Grant/Research Support|Melinta Therapeutics: Grant/Research Support|Menarini Ricerche S.p.A.: Grant/Research Support|Mutabilis: Grant/Research Support|Nabriva Therapeutics AG: Grant/Research Support|Paratek Pharmaceuticals, Inc.: Grant/Research Support|Qpex Biopharma: Grant/Research Support|Sfunga Therapeutics: Grant/Research Support|Spero Therapeutics: Grant/Research Support|Suzhou Sinovent Pharmaceuticals Co.: Grant/Research Support|Theravance: Grant/Research Support|tranScrip Partners: Grant/Research Support|University of Wisconsin: Grant/Research Support|Utility Therapeutics: Grant/Research Support|ValanBio Therapeutics Inc.: Grant/Research Support|VenatoRx: Grant/Research Support **Christopher M. Rubino, PharmD**, Adagio Therapeutics, Inc.: Grant/Research Support|Albany Medical Center: Grant/Research Support|Amplyx Pharmaceuticals, Inc.: Grant/Research Support|AN2 Therapeutics: Grant/Research Support|Antabio SAS: Grant/Research Support|Arcutis Biotherapeutics, Inc.: Grant/Research Support|B. Braun Medical Inc.: Grant/Research Support|Basilea Pharmaceutica: Grant/Research Support|BioFire Diagnostics LLC: Grant/Research Support|Boston Pharmaceuticals: Grant/Research Support|Cidara Therapeutics Inc.: Grant/Research Support|Cipla USA: Grant/Research Support|Crestone Inc.: Grant/Research Support|CXC: Grant/Research Support|Debiopharm International SA: Grant/Research Support|Entasis Therapeutics: Grant/Research Support|Genentech: Grant/Research Support|GlaxoSmithKline: Grant/Research Support|Hoffmann-La Roche: Grant/Research Support|ICPD: Ownership Interest|Inotrem: Grant/Research Support|Insmed Inc.: Grant/Research Support|Iterum Therapeutics Limited: Grant/Research Support|Kaizen Bioscience, Co.: Grant/Research Support|KBP Biosciences USA: Grant/Research Support|Matinas Biopharma: Grant/Research Support|Meiji Seika Pharma Co., Ltd.: Grant/Research Support|Melinta Therapeutics: Grant/Research Support|Menarini Ricerche S.p.A.: Grant/Research Support|Mutabilis: Grant/Research Support|Nabriva Therapeutics AG: Grant/Research Support|Paratek Pharmaceuticals, Inc.: Grant/Research Support|Qpex Biopharma: Grant/Research Support|Sfunga Therapeutics: Grant/Research Support|Spero Therapeutics: Grant/Research Support|Suzhou Sinovent Pharmaceuticals Co.: Grant/Research Support|Theravance: Grant/Research Support|tranScrip Partners: Grant/Research Support|University of Wisconsin: Grant/Research Support|Utility Therapeutics: Grant/Research Support|ValanBio Therapeutics Inc.: Grant/Research Support|VenatoRx: Grant/Research Support

